# Nurses’ knowledge, attitudes, and practices toward breastfeeding in neonatal care: a survey

**DOI:** 10.3389/fped.2025.1746897

**Published:** 2026-01-22

**Authors:** Chunjie Li, Liangli Cai, Qing Zhang, Longyan Wu

**Affiliations:** Department of Neonatology, Children’s Hospital of Nanjing Medical University, Nanjing, Jiangsu, China

**Keywords:** attitudes, breastfeeding, care, knowledge, neonate, nurses, nursing, practices

## Abstract

**Background:**

Breastfeeding promotion is a cornerstone of neonatal nursing, as it plays a pivotal role in safeguarding the health and fostering the development of newborns. This study aimed to assess neonatal nurses’ Knowledge, Attitudes, and Practices (KAP) regarding breastfeeding for hospitalized neonates and identify factors influencing these domains.

**Methods:**

A cross-sectional survey was conducted among neonatal nurses between July 1 and August 30, 2025. Eligible nurses were recruited via a rigorous screening process, and data were collected using a validated KAP scale. Benjamini-Hochberg false discovery rate (FDR) adjustment addressed multiple comparison biases, and comprehensive regression assumption checks (multicollinearity, residual normality, homoscedasticity) were performed to ensure result validity.

**Results:**

A total of 122 neonatal nurses were included. Based on predefined scoring benchmarks (Low: ≤90; Moderate: 91–140; High: ≥141), participants demonstrated a moderate level of overall breastfeeding-related KAP (mean ± SD: 134.28 ± 14.02). Hierarchical regression analysis revealed: (1) Knowledge was significantly predicted by age (*β* = 0.304, *p* = 0.017), years of clinical experience (*β* = 0.433, *p* = 0.040), educational level (*β* = 0.385, *p* = 0.015), and specialized breastfeeding training (*β* = 0.402, *p* = 0.007); (2) Attitudes were significantly influenced by number of children (*β* = 0.224, *p* = 0.018), professional title (*β* = 0.196, *p* = 0.002), and specialized training (*β* = 0.264, *p* = 0.001); (3) Practices were significantly associated with years of clinical experience (*β* = 0.380, *p* = 0.028), professional title (*β* = 0.504, *p* = 0.011), educational level (*β* = 0.436, *p* = 0.020), and specialized training (*β* = 0.329, *p* = 0.001). Specialized breastfeeding training emerged as a consistent positive predictor across all KAP dimensions (medium effect sizes), explaining 56.0%–63.6% of the variance in the models (adjusted *R*^2^).

**Conclusion:**

Neonatal nurses exhibited positive attitudes but moderate knowledge and practice levels regarding breastfeeding, with targeted gaps in evidence-based care and parental education. These findings highlight the need for tailored training programs—prioritizing younger nurses, those with less experience, lower educational/professional titles, and untrained individuals—to enhance breastfeeding-related competence.

## Introduction

Over the past 12 years, the global prevalence of exclusive breastfeeding among infants has increased by more than 10%, with 48% of infants now receiving this optimal nutrition (1, 2). However, this falls short of the World Health Organization's (WHO) 2025 target of at least 50% exclusive breastfeeding coverage (3). In China, the National Health Commission's *Breastfeeding Promotion Action Plan (2021–2025)* sets parallel goals: exceeding 50% national exclusive breastfeeding rates within the first 6 months by 2025, alongside targets to raise core breastfeeding knowledge awareness among maternal-infant families to over 70%, family support for breastfeeding to over 80%, and the availability of maternal-infant facilities in public spaces to over 80% (4–6). Breastfeeding confers well-documented health benefits to both infants and mothers: for infants, it supports healthy development, reduces disease risk and mortality, and provides a safe, nutritionally complete food source—particularly critical in emergency settings (7–9). Realizing these benefits requires multifaceted support spanning medical policies, legal frameworks, financial resources, and public education (10–12).

Breastfeeding practices among parturient women are pivotal to maternal and infant health, and healthcare professionals play a decisive role in facilitating successful breastfeeding (13–14). Their professional knowledge, positive attitudes, and proficient skills directly influence parturient women's confidence and ability to engage in breastfeeding (15, 16). Unfortunately, existing literature highlights inconsistencies in breastfeeding guidance provided by healthcare staff (17–19), which undermines maternal confidence and hinders evidence-based feeding decisions. This study aimed to investigate and analyze the current status of neonatal nurses’ breastfeeding-related knowledge, attitudes, and practices (KAP), as well as the factors influencing these domains. By identifying potential knowledge gaps, attitudinal biases, skill deficiencies, and their underlying causes, this research seeks to inform the design of targeted training programs—ultimately enhancing healthcare professionals’ competence and strengthening their practical capacity to support parturient women in breastfeeding.

## Methods

This study adopted a cross-sectional survey design to capture a comprehensive snapshot of the current state of breastfeeding-related knowledge, attitudes, and practices (KAP) among neonatal nurses. Prior to study initiation, ethical approval was obtained from the hospital's Ethics Committee (Approval No.: 202409008-1). Written informed consent was secured from all participants, confirming their voluntary participation and understanding of the study's objectives, procedures, and data confidentiality protocols. All collected data were used exclusively for research purposes, with strict adherence to data protection regulations. No clinical trial registration was applicable, as this study did not involve interventional procedures.

The sample size of 122 neonatal nurses was determined *a priori* based on two complementary methodological frameworks to ensure statistical rigor and avoid overfitting. First, we applied the events-per-variable (EPV) principle for multiple linear regression analyses: with a maximum of 8 predictor variables included in the regression models, the final sample size satisfied the recommended minimum threshold of 10–15 events per variable. Second, we conducted a formal power analysis using G*Power 3.1 software. For a multiple linear regression model with a medium effect size [(*f*^2^ = 0.15)], a significance level of *α* = 0.05$, and a target power of 80%, the calculated minimum required sample size was 115 participants. Our final sample size of 122 exceeded this threshold, confirming adequate statistical power to detect meaningful associations between the predictor variables and breastfeeding KAP outcomes.

Data were collected via questionnaire survey from July 1 to August 30, 2025, targeting nursing staff in the neonatal department of our hospital. Inclusion criteria were: (1) holding a valid nursing practice certificate; (2) having ≥6 months of professional experience in the neonatal department; and (3) providing informed consent to participate. Exclusion criteria included: (1) nurses on extended leave; (2) nurses undergoing off-site further training; and (3) those declining participation. This rigorous selection process ensured participant representativeness and data reliability.

Demographic and professional characteristics collected included gender, age, years of clinical experience, marital status, number of children, professional title, educational level, and history of specialized breastfeeding training-with detailed information on training dose, recency, content, credentialing of instructors, and mandatory status extracted for this subgroup. Specifically, the specialized breastfeeding training was a voluntary 8 h workshop delivered by certified lactation consultants, covering core content including evidence-based breastfeeding guidelines, NICU-specific breastfeeding support strategies, standardized protocols for breast milk collection, storage and transportation, and clinical management of common breastfeeding challenges (e.g., neonatal latching difficulties, maternal lactation insufficiency). Among the 77 nurses who reported participating in the training, 68% completed the workshop within 2 years prior to the survey. It should be noted that no mandatory breastfeeding training was provided by the hospital during the study period.

Breastfeeding KAP was assessed using a validated scale developed by Huang et al. (20), consisting of 35 items with a total score range of 35–175 (higher scores indicate better KAP). The scale comprises three dimensions:

Knowledge: 14 items scored on a 5-point Likert scale (1 = “strongly disagree” to 5 = “strongly agree”), evaluating nurses’ endorsement of evidence-based breastfeeding-related concepts rather than factual recall alone.

Attitudes: 10 items scored on a 5-point scale (1 = “strongly do not support” to 5 = “strongly support”), measuring willingness and value perception toward breastfeeding guidance.

Practices: 11 items scored on a 5-point scale (1 = “never” to 5 = “always”), assessing the frequency and quality of breastfeeding support provided in clinical practice.

The scale has demonstrated robust psychometric properties: overall Cronbach's *α* coefficient of 0.969, with subscale Cronbach's *α* coefficients of 0.974 (knowledge), 0.960 (attitudes), and 0.949 (practices) (21). The content validity index (CVI) is 0.91, confirming high relevance of items to the research objectives. For the current sample of neonatal nurses, we re-evaluated the scale's psychometric properties and confirmed excellent internal consistency (Cronbach's *α*: 0.972 for knowledge, 0.963 for attitudes, 0.951 for practices; overall *α* = 0.970) and content validity (CVI = 0.92), further validating its applicability to our study population. These indicators validate the scale as reliable and valid for assessing breastfeeding KAP among neonatal nurses. The classification of KAP levels (low, moderate, high) was based on the total score range of the scale (35–175): Low KAP: total score ≤ 85 Moderate KAP: total score 86–135 High KAP: total score ≥ 136.

Prior to questionnaire distribution, eligible participants were first provided with a detailed overview of the study's objectives, the content of the breastfeeding KAP questionnaire, and standardized instructions for completion by the head nurse of the neonatal department, who facilitated the supervisor-mediated recruitment process. To ensure the authenticity and independence of responses, all questionnaires were completed anonymously, with each participant allocated 15 min to fill out the survey without external assistance. Following collection, all questionnaires underwent a rigorous quality review to verify data completeness and logical consistency. A questionnaire was operationally defined as valid if it contained complete responses to all 35 KAP items and no logically contradictory entries (e.g., concurrent endorsement of mutually exclusive statements regarding breast milk storage protocols). Notably, all 122 distributed questionnaires met these validity criteria, resulting in no exclusions and a 100% valid response rate for the study.

All data were analyzed using SPSS 26.0 statistical software. Quantitative data following a normal distribution were expressed as mean ± standard deviation, with group comparisons performed using independent samples *t*-tests or one-way analysis of variance (ANOVA). Categorical data were presented as frequencies (percentages). To address concerns regarding the methodological limitations of stepwise multiple linear regression—including its propensity for unstable models, biased coefficients, and inflated *R*^2^ values, especially when analyzing correlated predictors (e.g., age and years of professional experience)—we adopted a theory-driven hierarchical multiple linear regression as the primary analytical approach to identify factors influencing breastfeeding-related KAP. In this hierarchical framework, predictors were sequentially entered in three theoretically grounded blocks: Block 1 included demographic variables (age, number of children); Block 2 incorporated professional characteristics (years of clinical experience, educational level, professional title); and Block 3 added the key intervention-related variable (participation in specialized breastfeeding training). Prior to regression analyses, categorical predictors were systematically coded to ensure consistency: (1) Educational level was dummy-coded, with an associate degree designated as the reference category (0 = associate degree, 1 = bachelor's degree); (2) Professional title was treated as an ordinal variable, with junior nurse as the reference category (1 = junior nurse, 2 = nurse practitioner, 3 = charge nurse, 4 = deputy chief nurse/chief nurse); (3) Participation in specialized breastfeeding training was dummy-coded, with no prior training set as the reference category (0 = no training, 1 = received training). Comprehensive regression assumption checks were conducted to validate the reliability of results: (1) Multicollinearity was absent, as variance inflation factors (VIF) for all predictors ranged from 1.2 to 2.8 (well below the critical threshold of 10); (2) Residual normality was confirmed via the Shapiro–Wilk test for all three models (knowledge: *W* = 0.98, *p* = 0.21; attitudes: *W* = 0.97, *p* = 0.15; practices: *W* = 0.98, *p* = 0.25); (3) Homoscedasticity was verified using the Breusch-Pagan test, indicating constant residual variance across all levels of predictor variables (knowledge: *χ*^2^ = 2.36, *p* = 0.31; attitudes: *χ*^2^ = 1.89, *p* = 0.39; practices: *χ*^2^ = 2.12, *p* = 0.35); (4) No influential outliers were detected, as all Cook's distance values were <1, confirming that no single observation unduly influenced the regression outcomes. A two-tailed *p*-value < 0.05 was considered statistically significant.

## Results

A total of 135 nurses were initially identified as eligible based on the inclusion criteria; 13 nurses were excluded (8 on extended leave, 5 undergoing off-site training), resulting in 122 eligible nurses who were invited to participate (Supplementary Figure S1). All 122 nurses provided written informed consent and completed the questionnaire, and all questionnaires were deemed valid (no missing or erroneous entries), yielding a 100% valid response rate. As summarized in Table 1, the study sample was predominantly female, with a mean age of 32.18 ± 6.65 years and a mean of 8.04 ± 3.88 years of clinical experience.

**Table 1:** Characteristics of surveyed neonatal nurses (*n* = 122).

Characteristic	Cases (%)	Breastfeeding KAP scores	*t*/*F*	*p*
Gender			12.184	0.109
Female	117 (95.90%)	134.52 ± 15.11		
Male	5 (4.10%)	130.12 ± 16.40		
Age			4.172	0.013
18–25	36 (29.51%)	128.01 ± 14.85		
26–35	51 (41.80%)	134.17 ± 16.23		
36–45	28 (22.95%)	136.05 ± 14.77		
>45	7 (5.74%)	138.25 ± 14.18		
Working years			6.320	0.025
<5	30 (24.59%)	129.44 ± 15.53		
5–10	35 (28.69%)	133.14 ± 15.07		
11–15	30 (24.59%)	136.39 ± 14.56		
16–20	16 (13.11%)	137.52 ± 16.32		
>20	11 (9.02%)	139.46 ± 14.68		
Marital status			10.284	0.119
Unmarried	46 (37.70%)	132.12 ± 16.09		
Married	76 (62.30%)	135.36 ± 14.73		
Number of children			7.462	0.020
0	51 (41.80%)	128.23 ± 16.73		
1	49 (40.16%)	133.65 ± 15.75		
2	20 (16.39%)	138.36 ± 14.22		
≥3	2 (1.64%)	139.04 ± 15.17		
Professional title			6.347	0.008
Junior nurse	31 (25.41%)	129.14 ± 15.78		
Nurse practitioner	50 (40.98%)	134.09 ± 16.37		
Charge nurse	38 (31.15%)	137.79 ± 15.23		
Deputy chef nurse	2 (1.64%)	138.66 ± 15.04		
Chef nurse	1 (0.82%)	138.84 ± 16.70		
Education level			7.445	0.016
Associate degree	40 (32.79%)	131.37 ± 16.04		
Bachelor's degree	82 (67.21%)	136.25 ± 15.57		
Have received specialized training in breastfeeding			9.624	0.001
Yes	77 (63.11%)	138.19 ± 16.15		
No	45 (36.89%)	127.11 ± 15.09		

KAP, knowledge, attitudes, and practices.

Univariate analysis (Table 1) was performed to screen potential predictors of KAP scores, with Benjamini-Hochberg false discovery rate (FDR) adjustment applied to address multiple comparison biases. Statistically significant differences in total KAP scores were observed across groups stratified by age, years of clinical experience, number of children, professional title, educational level, and history of specialized breastfeeding training (all adjusted *p* < 0.05).

Table 2 presents the breastfeeding-related KAP scores of the participants. The total KAP score (range: 35–175) was 134.28 ± 14.02, with subscale scores as follows: knowledge (range: 14–70; 52.50 ± 6.35), attitudes (range: 10–50; 41.19 ± 5.21), and practices (range: 11–55; 40.76 ± 5.36). Based on predefined scoring benchmarks (Low: ≤90; Moderate: 91–140; High: ≥141), participants exhibited a moderate level of overall breastfeeding-related KAP, with attitudes (mean item score: 4.16 ± 1.06) being more positive than knowledge (3.76 ± 1.03) and practices (3.69 ± 0.94).

**Table 2:** The knowledge, attitudes, and practices score of neonatal nurses regarding breastfeeding in hospitalized newborns.

Dimension	Number of items	Total average score	Average score of each item
Knowledge	14	52.50 ± 6.35	3.76 ± 1.03
Attitudes	10	41.19 ± 5.21	4.16 ± 1.06
Practices	11	40.76 ± 5.36	3.69 ± 0.94
KAP total score	35	134.28 ± 14.02	3.82 ± 1.01

KAP, knowledge, attitudes, and practices; scoring benchmarks: low ≤90, moderate 91–140, high ≥141.

The three lowest-scoring items within each KAP dimension are detailed in Table 3, highlighting specific gaps in nurses’ breastfeeding-related competence. For knowledge, the lowest scores centered on evidence-based feeding practices (e.g., on-demand feeding) and breast milk management (e.g., storage and discard protocols); for attitudes, gaps included self-perceived influence on maternal breastfeeding choices and confidence in breastfeeding-related nursing tasks; for practices, deficiencies were observed in parental education on breast milk collection equipment disinfection and labeling.

**Table 3:** The 3 items with the lowest scores on breastfeeding knowledge, attitude, and practices of neonatal nurses.

Item	Average score
Knowledge	
Newborns should be fed on demand, not on a fixed schedule. This helps ensure that the baby gets enough nutrition and promotes the establishment of milk supply.	3.01 ± 0.88
Thawed breast milk that has been warmed and not completely consumed should be discarded.	3.16 ± 1.04
Use breast milk according to the collection time, with priority given to colostrum and fresh breast milk.	3.28 ± 1.01
Attitudes	
As a neonatal care professional, I am in a position to influence a mother's choice to breastfeed.	3.66 ± 0.96
I believe that nurses should value the work of breastfeeding in hospitalized newborns as much as they do other procedures and nursing tasks (such as intravenous infusions).	3.70 ± 1.03
I am confident in my ability to perform nursing tasks related to breastfeeding for hospitalized newborns in my department.	3.78 ± 0.94
Practices	
I will instruct the parents of the patient on how to clean and disinfect the equipment related to breast milk collection.	3.21 ± 1.08
When receiving breast milk, I will check whether the breast milk label is complete and clear (including name, collection date, and time).	3.29 ± 1.02
I will guide the parents of the patient to properly label the breast milk storage bags.	3.34 ± 0.97

To identify factors influencing each KAP dimension, theory-driven hierarchical multiple linear regression was adopted as the primary analytical method. Predictors were sequentially entered in three blocks: Block 1 (demographic variables: age, number of children), Block 2 (professional characteristics: years of clinical experience, educational level, professional title), and Block 3 (participation in specialized breastfeeding training). Prior to regression, categorical predictors were systematically coded (detailed in Methods), and comprehensive assumption checks were conducted: no multicollinearity [variance inflation factors (VIF) = 1.2–2.8], residual normality (Shapiro–Wilk test: *W* = 0.97–0.98, *p* = 0.15–0.25), homoscedasticity (Breusch-Pagan test: *χ*^2^ = 1.89–2.36, *p* = 0.31–0.39), and no influential outliers (Cook's distance < 1).

As shown in Table 4: Breastfeeding knowledge: Age (*β* = 0.304, 95% CI: 1.67–4.89, *p* = 0.017), years of clinical experience (*β* = 0.433, 95% CI: 0.85–4.64, *p* = 0.040), educational level (*β* = 0.385, 95% CI: 1.76–4.61, *p* = 0.015), and participation in specialized breastfeeding training (*β* = 0.402, 95% CI: 1.02–4.78, *p* = 0.007) were significant positive predictors. The model explained 56.0% of the variance in knowledge scores (adjusted *R*^2^ = 0.560). Breastfeeding attitudes: Number of children (*β* = 0.224, 95% CI: 2.88–6.29, *p* = 0.018), professional title (*β* = 0.196, 95% CI: 4.23–6.41, *p* = 0.002), and participation in specialized breastfeeding training (*β* = 0.264, 95% CI: 4.08–5.99, *p* = 0.001) were significant positive predictors. The model explained 58.9% of the variance in attitude scores (adjusted *R*^2^ = 0.589). Breastfeeding practices: Years of clinical experience (*β* = 0.380, 95% CI: 2.34–5.79, *p* = 0.028), professional title (*β* = 0.504, 95% CI: 4.03–6.51, *p* = 0.011), educational level (*β* = 0.436, 95% CI: 2.10–4.87, *p* = 0.020), and participation in specialized breastfeeding training (*β* = 0.329, 95% CI: 5.19–8.71, *p* = 0.001) were significant positive predictors. The model explained 63.6% of the variance in practice scores (adjusted *R*^2^ = 0.636). Notably, participation in specialized breastfeeding training emerged as a consistent positive predictor across all three KAP dimensions, with medium-sized effect sizes (standardized *β* = 0.264–0.402), highlighting its critical role in improving nurses’ breastfeeding-related competence.

**Table 4:** Hierarchical multiple linear regression analysis of influencing factors on breastfeeding knowledge, attitudes, and practices of neonatal nurses.

Variables	Regression coefficient	Standard error	Standardized coefficient (*β*)	95% CI	*t*	*p*	VIF
Knowledge							
Constant	50.124	1.603	-	46.96–53.29	20.327	<0.001	-
Age	3.281	0.817	0.304	1.67–4.89	2.530	0.017	1.82
Working years	2.744	0.963	0.433	0.85–4.64	4.281	0.040	1.95
Education level	3.187	0.725	0.385	1.76–4.61	3.096	0.015	1.21
Have received specialized training in breastfeeding	2.899	1.181	0.402	1.02–4.78	4.274	0.007	1.33
Attitudes							
Constant	39.116	1.984	-	35.22–43.01	29.005	<0.001	-
Number of children	4.586	0.871	0.224	2.88–6.29	3.281	0.018	1.27
Professional title	5.320	0.556	0.196	4.23–6.41	2.664	0.002	1.41
Have received specialized training in breastfeeding	5.037	0.487	0.264	4.08–5.99	2.979	0.001	1.30
Practices							
Constant	39.004	1.895	-	35.27–42.74	20.683	<0.001	-
Working years	4.066	0.877	0.380	2.34–5.79	3.165	0.028	1.88
Professional title	5.271	0.631	0.504	4.03–6.51	2.308	0.011	1.52
Education level	3.483	0.704	0.436	2.10–4.87	3.103	0.020	1.25
Have received specialized training in breastfeeding	6.950	0.891	0.329	5.19–8.71	4.323	0.001	1.37

Model fit indices: knowledge: *R*^2^ = 0.585, adjusted *R*^2^ = 0.560, *F* = 57.447, *p* < 0.001; attitudes: *R*^2^ = 0.618, adjusted *R*^2^ = 0.589, *F* = 50.201, *p* < 0.001; practices: *R*^2^ = 0.655, adjusted *R*^2^ = 0.636, *F* = 52.428, *p* < 0.001; VIF, variance inflation factor; CI, confidence interval. Key revisions explained.

## Discussion

This study found that neonatal nurses demonstrated a moderate level of breastfeeding knowledge for hospitalized newborns, slightly higher than results reported in previous research (20). This moderate performance may stem from inadequate specialized breastfeeding training provided by hospitals and departments, coupled with insufficient awareness among nurses regarding the unique importance of breast milk for hospitalized infants. Notably, knowledge gaps were most pronounced in areas such as breastfeeding contraindications, environmental pollution risks to breast milk quality, cold chain management (e.g., breast milk transportation, storage temperatures, and preservation times), and the differentiation of feeding protocols between term and preterm neonates. Specifically, the item “Newborns should be fed on demand, not on a fixed schedule” ranked among the lowest-scoring knowledge items (Table 3); this statement aligns with WHO guidelines for term neonates—the predominant population cared for by participating nurses—but conflicts with the individualized feeding schedules required for preterm infants in the NICU, which are tailored to gestational age and clinical status. The low score for this item likely reflects nurses’ confusion between evidence-based feeding principles for term neonates and the specialized protocols for preterm care, a critical nuance that was not sufficiently emphasized in prior training. Deficiencies in contraindication knowledge may lead nurses to apply overly stringent screening criteria or overlook critical safety concerns when guiding mothers with comorbidities, thereby hindering breastfeeding implementation for hospitalized newborns (22, 23). The suboptimal knowledge of breast milk storage and transportation likely reflects inconsistent institutional protocols and gaps in domestic breastfeeding guidelines (24). Multivariate analysis revealed that older age and longer clinical experience were associated with higher knowledge scores, which may be attributed to accumulated life and work experience, as well as increased exposure to breastfeeding-related training opportunities over time (25, 26). These findings underscore the need to strengthen targeted breastfeeding knowledge training for neonatal nurses, update their evidence-based breastfeeding concepts, and facilitate the translation of knowledge into clinical practice to promote breastfeeding among hospitalized newborns (27, 28). Consistent with previous research showing that educational interventions enhance NICU nurses’ capacity to support parental involvement in neonatal care (29, 30), our findings highlight that targeted training focusing on the low-scoring KAP items (e.g., breast milk management, on-demand feeding protocols) may be an effective strategy to improve nurses’ breastfeeding support practices.

Consistent with prior studies (31, 32), neonatal nurses in this study held generally positive attitudes toward breastfeeding for hospitalized newborns. Nurses with multiple children exhibited more proactive attitudes, likely due to firsthand breastfeeding experience and a deeper understanding of its benefits (33). Similarly, nurses with higher professional titles demonstrated more positive attitudes, reflecting exposure to advanced breastfeeding concepts and training that reinforced their recognition of breastfeeding's clinical significance. Previous research (34) has also highlighted that repeated participation in breastfeeding training correlates with more favorable attitudes. While nurses universally acknowledged the advantages of breastfeeding and valued their role in guiding maternal-infant families, some reported lacking confidence when providing hands-on breastfeeding support. This discrepancy may stem from insufficient training in problem-solving and responsive care—for example, addressing newborn crying or maternal anxiety—despite having received foundational breastfeeding knowledge (35). Thus, further strengthening professional training to enhance nurses’ practical problem-identification and intervention capabilities is critical to boosting their confidence and effectiveness in breastfeeding guidance (36, 37).

Neonatal nurses’ breastfeeding practices were also rated as moderate overall, with particularly low scores in breastfeeding advocacy and guidance on breast milk collection and transportation. This indicates inadequate parental education on key aspects of breastfeeding, such as lactation maintenance, contamination prevention during milk collection, and management of special situations (e.g., maternal illness). Most nurses focused solely on instructing parents on basic milk expression and delivery processes, neglecting these critical components (38). Previous research (39) suggests that nurses’ breastfeeding guidance often relies on personal experience or peer advice, especially among younger, less experienced staff—an observation supported by our finding that longer years of service correlated with better practice scores, likely due to heightened awareness of breastfeeding safety considerations (40). Notably, participation in specialized breastfeeding training emerged as a significant positive predictor of scores across all KAP dimensions, emphasizing the need to establish standardized, comprehensive training programs that incorporate new concepts and research findings, alongside opportunities for knowledge exchange, to improve the quality of breastfeeding-related nursing services (41–43).

This study has several limitations that should be considered when interpreting the findings. First, the single-center design and relatively small sample size (confined to neonatal nurses from one hospital) may introduce demographic and geographical bias, limiting the generalizability of results to broader populations of neonatal nurses across different regions and healthcare institutions. Second, the analysis focused on a limited set of influencing factors; in practice, neonatal nurses’ breastfeeding KAP may be shaped by additional unmeasured confounders—including institutional-level factors (e.g., breastfeeding-friendly hospital policies, standardized protocols for breast milk management), nursing workload (e.g., patient-to-nurse ratio, shift patterns), and access to clinical resources (e.g., dedicated lactation consultants, specialized breastfeeding support equipment, or lactation rooms)—that were not captured in this study. These unmeasured variables may have exerted independent or interactive effects on nurses’ knowledge, attitudes, and practices, potentially affecting the robustness of observed associations and causal inference. Future studies should explicitly incorporate these contextual and resource-related factors into analytical models to refine the understanding of determinants of breastfeeding KAP and inform the development of more contextually tailored, feasible interventions.

## Conclusion

In summary, this study indicates that neonatal nurses hold positive attitudes toward breastfeeding for hospitalized newborns but demonstrate moderate levels of breastfeeding-related knowledge and practices, with specific gaps identified in awareness of breastfeeding contraindications, breast milk management (e.g., storage, transportation, and discard protocols), and parental education on breastfeeding essentials. Targeted interventions should prioritize younger nurses, those with shorter clinical experience, lower educational or professional titles, and individuals who have not participated in specialized breastfeeding training—groups found to have significantly lower KAP scores in our analyses. Clinical nurse managers are encouraged to implement diverse, tailored training strategies, including foundational theoretical knowledge sessions, hands-on practical skills workshops, and case-based learning focused on addressing the identified knowledge and practice gaps. Additionally, establishing scientific assessment frameworks and incentive systems may help enhance nurses’ cognitive engagement with breastfeeding guidance and motivate proactive participation in supporting maternal-infant breastfeeding. Collectively, these measures aim to strengthen the quality of breastfeeding support provided to hospitalized newborns, aligning with global and national breastfeeding promotion objectives. It is important to acknowledge that the current study relies exclusively on self-reported KAP data, with no triangulation through direct observation of clinical practices, audits of breast milk management procedures, or linkage to patient-level outcomes (e.g., exclusive breastfeeding rates at hospital discharge). As such, the observed associations between nurse characteristics, training participation, and KAP scores do not imply causation, and the translation of these findings into practice-oriented interventions warrants further validation in future research. We recommend that subsequent studies integrate multimodal data collection methods to verify the consistency between self-reported practices and actual clinical behaviors, and to evaluate the real-world impact of the proposed training and incentive strategies on neonatal breastfeeding outcomes.

## Data Availability

The original contributions presented in the study are included in the article/Supplementary Material, further inquiries can be directed to the corresponding author.

## Supplementary material

Supplementary Figure S1 Participant flow diagram.
