# Supplementary figures and images for: Nurses’ knowledge, attitudes, and practices toward breastfeeding in neonatal care: a survey
Source: Front Pediatr. 2026 Jan 22;13:1746897. doi: 10.3389/fped.2025.1746897 (PMC12872813; doi:10.3389/fped.2025.1746897)

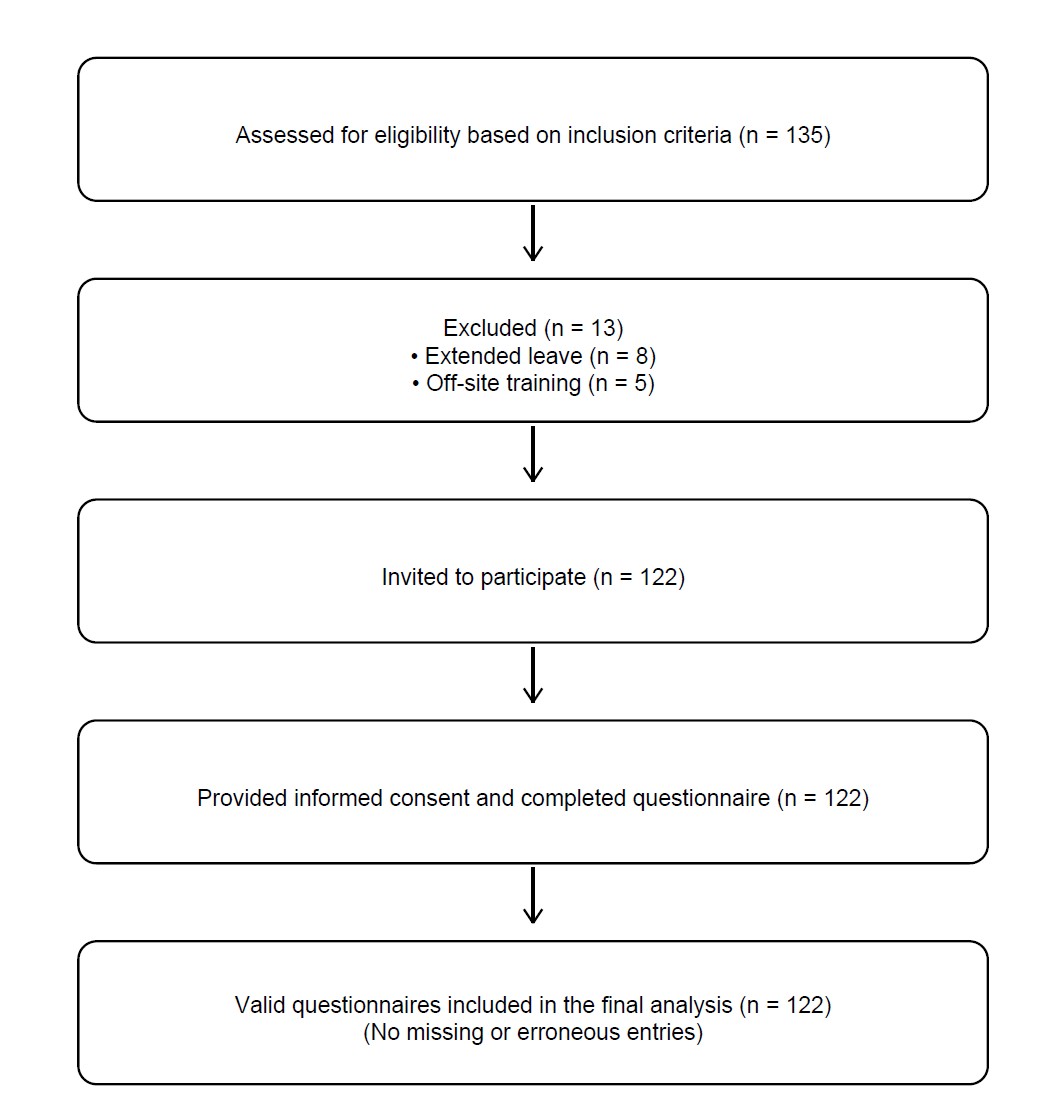

Supplement: Supplementary Figure S1 — Participant flow diagram. [file Image1.jpeg]
